# Repurposing Antibacterial AM404 As a Potential Anticancer Drug for Targeting Colorectal Cancer Stem-Like Cells

**DOI:** 10.3390/cancers12010106

**Published:** 2019-12-31

**Authors:** Mehreen Ahmed, Nicholas Jinks, Roya Babaei-Jadidi, Hossein Kashfi, Marcos Castellanos-Uribe, Sean T. May, Abhik Mukherjee, Abdolrahman S. Nateri

**Affiliations:** 1 Cancer Genetics & Stem Cell Group, BioDiscovery Institute, Division of Cancer and Stem Cells, School of Medicine, University of Nottingham, Nottingham NG7 2UH, UK; mehreen.ahmed@nottingham.ac.uk (M.A.); msxnj@exmail.nottingham.ac.uk (N.J.); mszrb3@exmail.nottingham.ac.uk (R.B.-J.); mzxsmk@exmail.nottingham.ac.uk (H.K.); 2 Respiratory Medicine, School of Medicine, University of Nottingham, Nottingham NG7 2UH, UK; 3 Nottingham Arabidopsis Stock Centre (NASC), Plant Science Building, School of Biosciences, University of Nottingham, Loughborough LE12 5RD, UK; sbzmc3@exmail.nottingham.ac.uk (M.C.-U.); sbzstm@exmail.nottingham.ac.uk (S.T.M.); 4 Department of Histopathology, Queen’s Medical Centre, School of Medicine, University of Nottingham, Nottingham NG7 2UH, UK; mszam2@exmail.nottingham.ac.uk

**Keywords:** AM404, cancer stem cells, colonosphere, CRC, differentiation, drug screening, FBXL5 E3-ligase, patient derived organoids, resistance and metastasis, tissue explants

## Abstract

Tumour-promoting inflammation is involved in colorectal cancer (CRC) development and therapeutic resistance. However, the antibiotics and antibacterial drugs and signalling that regulate the potency of anticancer treatment upon forced differentiation of cancer stem-like cell (CSC) are not fully defined yet. We screened an NIH-clinical collection of the small-molecule compound library of antibacterial/anti-inflammatory agents that identified potential candidate drugs targeting CRC-SC for differentiation. Selected compounds were validated in both in vitro organoids and ex vivo colon explant models for their differentiation induction, impediment on neoplastic cell growth, and to elucidate the mechanism of their anticancer activity. We initially focused on AM404, an anandamide uptake inhibitor. AM404 is a metabolite of acetaminophen with antibacterial activity, which showed high potential in preventing CRC-SC features, such as stemness/de-differentiation, migration and drug-resistance. Furthermore, AM404 suppressed the expression of *FBXL5* E3-ligase, where AM404 sensitivity was mimicked by *FBXL5*-knockout. This study uncovers a new molecular mechanism for AM404-altering FBXL5 oncogene which mediates chemo-resistance and CRC invasion, thereby proposes to repurpose antibacterial AM404 as an anticancer agent.

## 1. Introduction

Colorectal cancer (CRC) is the fourth most common cancer and leads to approximately 500,000 deaths a year worldwide [1]. For patients with CRC, chemotherapy remains the most common treatment but often followed by tumour regrowth due to acquired chemotherapy-resistance. Experimental evidence supports the role of small fraction of cancer stem-like cells (CSCs) in the tumour, including colorectal cancer [2,3,4]. CSCs exhibit several distinctive features such as enhanced self-renewal and limited differentiation capacity that allow them to be resistant to anti-cancer therapies and tumour-targeted drugs, which in turn, helps them to survive treatment and initiate tumour recurrence [5]. However, selective targeting of CSC is a huge challenge from the therapeutic point of view as strategies not being sufficiently selective for CSCs also increase risks of recurrence among the patients [6]. Therefore, there is an ever-growing need for novel compounds and drugs that target CSCs, preferably in combination with other cytotoxic drugs and tumour-targeted agents to prevent the regrowth of neoplastic cell populations.

Several multidimensional approaches have been utilized to target specific markers or pathways to eliminate CSCs, alter tumour microenvironment, induce differentiation, re-sensitization to chemotherapy, apoptosis, and reversal of epithelial-mesenchymal transition (EMT) [7,8]. Nevertheless, the association of stem cell signatures with disease outcome in several types of cancer is widely established. Due to the characteristic features of cancer and their association with other diseases, it is also important to investigate these cancer traits in order to target them. For example, inflammation is one of the major hallmarks of cancer [9], as evident at the earliest stages of cancer progression and is capable of fostering growth of small tumors into metastatic cancers [10]. In addition, some FDA approved commercially available antibacterial and anti-inflammatory drugs are currently being studied for their potential in targeting CSCs. Such examples include salinomycin, curcumin, metformin, vismodegib, EGCG, imetelstat, heparin, resveratrol, tranilast, amongst others [11,12,13,14]. Therefore, investigating the parameters and drugs greatly benefits the study of cancer therapeutics. Based on this hypothesis, we carried out drug screening on a library of 707 FDA (the Food and Drug Administration) approved small molecule compounds with anti-inflammatory, antibacterial activities using 3D colonosphere as a model representing CRC-SC [15,16]. We have validated and used stem cell fluorescent probe (i.e., CDy1) as a reporter for the rapid screening of compounds in their differentiation-inducing potential [17,18]. Our further analysis of patient-derived tumour explant and organoid models and the molecular mechanisms data has identified antibacterial AM404 as a potential candidate for targeting CRC stem-like cells.

**Figure 1:**
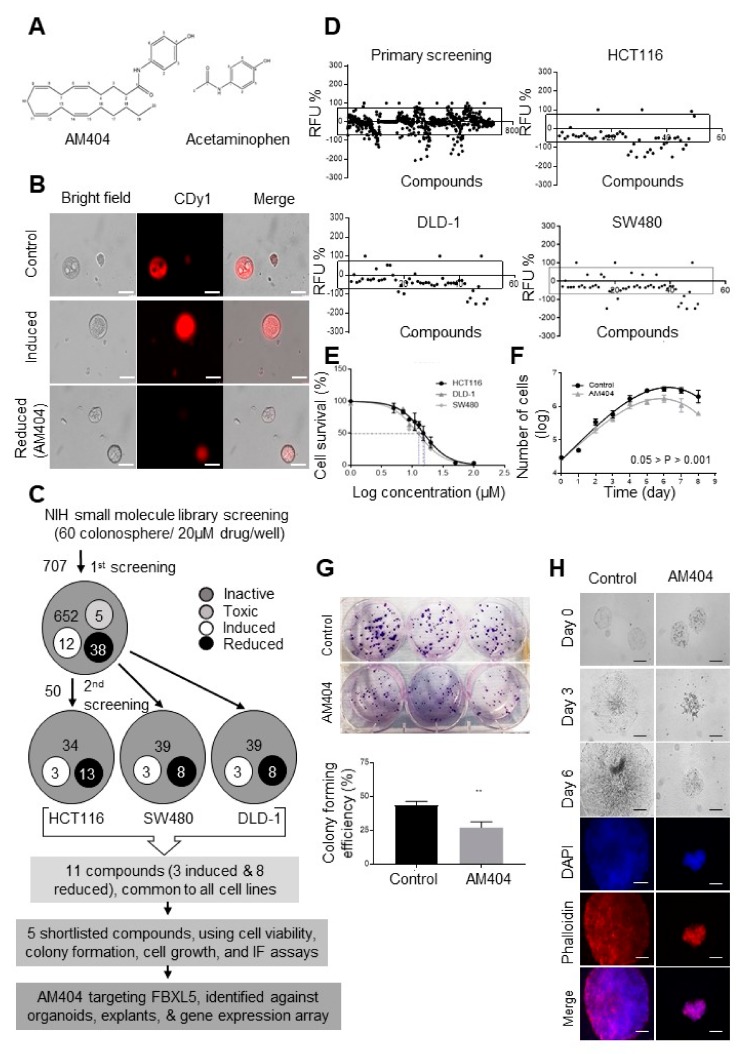
Screening of NIH library containing 707 small-molecule inhibitors, can induce 3D colonosphere differentiation. (**A**) Structural formula of AM404 and acetaminophen. (**B**) Representative images of the fluorescence intensity conferred by CDy1 on compounds and control-treated colonospheres obtained from HCT116 cells. Top row represents untreated colonospheres intensity, whereas middle and bottom row represent induced and reduced intensity as measure of induce and reduced stem-like characteristics upon treatments. Scale bar: 25 µM. (**C**) Summary of screening. (**D**) Primary screening based on fluorescence intensity influenced by small molecules on colonospheres derived from HCT116 cells. Each dot represents one compound (*n* = 2). All compound treated colonosphere intensities were expressed as percentage of the control-treated intensities as an indication of induced or reduced stemness. Compounds outside the square-zone were selected for a rescreening. At the end of rescreening (*n* = 3), 11 common compounds from 3 cell lines were selected based on their potential on CDy1 intensity induction and/or reduction. (**E**) IC_50_ of AM404 in HCT116, DLD-1 and SW480 cell lines. IC_50_ was measured at 15.2, 15.3 and 12.3 µM respectively. (**F**) Growth curve of AM404-treated DLD-1 cells. Student’s *t*-test was performed for the statistical analysis. Error bars represent mean ± S.D. (*n* = 3). 0.05 > *p* > 0.001. (**G**,**H**) AM404 showing morphological alteration and significant reduction in colony formation assay in DLD-1 cell line. ** *p* ≤ 0.01. Scale bar: 75 µm.

AM404 is a metabolite of acetaminophen with antibacterial activity, also known as N-arachidonoylphenolamine with a chemical formula of C_26_H_37_NO_2_ [19]. Acetaminophen (N-acetyl-*para*-aminophenol or paracetamol) is one of the most commonly used over-the-counter drugs for its analgesic and antipyretic properties [20]. Following its administration, AM404 has been reported in human cerebrospinal fluid [21]. Acetaminophen undergoes de-acetylation to p-aminophenol in both liver and nervous system and p-aminophenol is conjugated with arachidonic acid to produce AM404 in the nervous system. It has been suggested that AM404 may be responsible for the analgesic mechanism of paracetamol [19]. Some studies have demonstrated AM404s antibacterial and anti-inflammatory effects in reducing oxidative stress are associated with the presence of the phenolic group in its structure (Figure 1A) [19,22,23]. Currently, there is no established data available for AM404 in colorectal cancer. Our data suggest that ubiquitin-ligase FBXL5 [24,25,26,27], might be a key target through which AM404 utilizes its pharmacological effects on CRC cells.

## 2. Results

### 2.1. A Screen of the NIH Clinical Collection Small Molecule Library Identifies Potential Anti-Cancer Drug AM404

The 3D colonospheres were obtained from HCT116, DLD-1 and SW480 human CRC cell lines according to their colonosphere forming efficiencies and were employed into a fluorescence-based screening of US National Institute of Health (NIH) clinical library consisting of 707 small molecule inhibitors (Figure S1). One particular advantage of this screening was that it has been carried out on live colonospheres without any fixation step involved. Prior to the compound library screening, we initially carried out a pre-screening study with stem cell dye CDy1 using a HDAC inhibitor and *FBXW7* deleted CRC cells (Figure S1 and Table S1). Vorinostat (SAHA) is a potent HDAC inhibitor that has previously been reported to induce differentiation and has undergone Phase I and II clinical trials [28,29,30]. On the other hand, our lab and others have reported FBXW7 as one of the most frequently mutated genes in CRC, and have associated its loss with chromosomal instability, cellular proliferation, EMT, and overall tumorigenesis [31,32,33,34]. In order to carry out the pilot-screening, we incorporated both vorinostat treatment (to induce differentiation) and HCT116^FBXW7(−/−)^ derived colonospheres (to represent high tumorigenesis), within the CDy1 based screening system. 

Our results showed CDy1 intensities were significantly reduced in vorinostat-treated colonospheres, whereas, it was induced in HCT116^FBXW7(−/−)^ derived colonospheres, further demonstrating successful use of CDy1 as an indicator of stemness/differentiation induction. Based on the pre-screening, well defined colonospheres derived from HCT116 cells were collected carefully with mild agitation and ensured of uniform transfer (~60 colonospheres/well) in 96 well plates. Colonospheres were then treated with 707 compounds (at final concentration of 20 μM) for 72 h before selectively stain the live stem cells, as magnitude of drug-induced stemness and/or differentiation level represented by high and low CDy1 fluorescence intensity respectively. HCT116 cells were primarily chosen for the initial screening based on their highly aggressive, resistant and non-differentiating nature [35]. The concentration of compounds was selected based on previous studies being carried out at 10 μM in monolayer cells, in line with results from our lab showing significantly higher resistance with 3D colonospheres than 2D cells [5,33]. Initial screening identified 50 compounds based on distinct morphology changes, colonosphere sizes and CDy1 intensity (Figure 1B–D and Table S2). Next, we carried out a re-screening using other CRC cell lines (SW480 and DLD-1), in addition to HCT116 cells (Figure 1D) that identified 11 compounds for their ability in inducing and/or reducing stem-like prowess (Table S2). Amongst the compounds that reduced the stem-like characteristics, more recent work showed that the antifungal drug itraconazole targets cell cycle heterogeneity, and epirubicin targets metastasis and DNA-damage induced-drugs resistance in CRC [36,37]. However, the SRB assay was used for over a wide range of doses (1 to 100 μM) to calculate the half-maximal inhibitory concentration (IC50) which defined AM404 as a better candidate with an IC_50_ that is lower than the target threshold (20 μM) for further in-depth evaluations [5]. This result was backed by the previous studies reporting AM404 to be well tolerated on animal models and being less toxic on mammalian cells including human HEK-293, HepG2, and, Panc-1 cells for up to the 4X of the MIC, indicating its relatively safe profile [22,23]. Our results were highly comparable between the DLD-1 and HCT116 cell lines, with IC50 of 15.3 and 15.2 µM respectively, whereas, AM404 shows slightly more sensitivity towards SW480 with an IC_50_ of 12.3 µM (Figure 1E). Next, cells were treated with the IC_50_ of AM404 (Figure 1E) on day 1 and were counted every day for a period of 8 days. AM404-treated DLD-1 cells showed a shift in the population doubling time (PDT) from 21 h to 29 h as shown in control-treated cells. This lag in the doubling time indicates significant impedance on cell growth (Figure 1F). Our results showed a significant reduction in the number of colonies during the treatment along with their morphological alteration upon AM404 treatment (Figure 1G). Reduction in colony size was strongly evident within the first three days of treatment, which was also seen in the growth curve (Figure 1G,H). Phalloidin staining significantly distinguishes the morphological differences conferred by AM404 in DLD-1-treated colonies (Figure 1H). Next, we examined the effect of AM404 on the sensitivity to the drugs 5-fluorouracil (5-FUra) and oxaliplatin (Oxa) that are widely used for cancer treatment, particularly for CRC [38]. Also, we have previously investigated the sensitivity to 5-FUra and Oxa drugs and showed that FBXW7-deficiency-induced chemoresistance [33,39]. 

**Figure 2:**
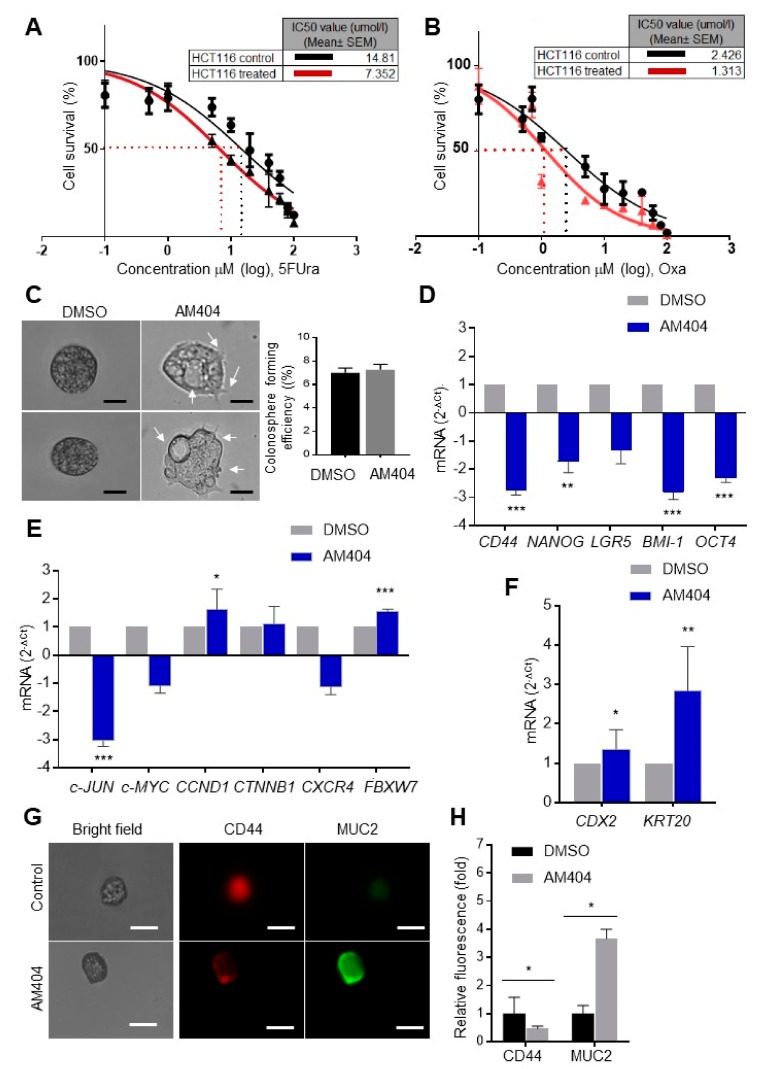
AM404 association with stem-like characterizes and differentiation. (**A**,**B**) Survival of synchronized/ serum-starved FBXW7-deficient HCT116 cell lines (HCT116^FBXW7(−/−)^). Black colour indicates treatment with 10 increasing concentrations of 5-FU (**A**) and Oxaliplatin (**B**), whereas red indicates co-treatment with AM404. SRB colorimetric assay was performed in triplicate for each cell line on three independent occasions. IC_50_ values, calculated by using GraphPad Prism software 7.02, represent the mean of three different experiments ± SEM with *p* ≤ 0.005. Cells co-treated with AM404 are found to be more sensitive to 5-FU (**A**) with an IC_50_ of 7.35 vs. 14.8 µM and to Oxaliplatin (**B**) with an IC_50_ of 1.3 vs. 2.4 µM. (**C**) AM404 showing morphological alteration in colonosphere with no change in colonosphere formation efficiency. Scale bar: 25 µM. (**D**–**F**) qRT-PCR analysis of CRC-SC (**D**), stemness (**E**), transcription factor for recurrence, poor survival, metastasis and tumour suppressor, and (**F**) differentiation in DLD-1 derived colonospheres treated with AM404. Student’s *t*-test was performed for the statistical analysis. *, *p* ≤ 0.05; **, *p* ≤ 0.01; ***, *p* ≤ 0.001. (**G**) Immunofluorescence assay of AM404 treated colonospheres using stemness and differentiation markers, Scale bars: 25 µm. (**H**) AM404 treatment shows increased MUC2 expression and reduced level of CD44 expression suggesting induction of differentiation upon drug treatment (right panel, *n*  =  15, *p* ≤ 0.05).

Our results indicated that HCT116^FBXW7(−/−)^ cells treated with AM404 were more sensitive to treatment with these drugs, following synergistic effects with CI < 1 (Figure 2A,B, Figure S2) [40,41]. These results further confirmed AM404 as a potential anticancer drug candidate in CRC cells.

### 2.2. AM404 Inhibits De-Differentiation and Acquisition of Stem-Like Properties

The relative changes in colonospheres size/shape were assessed by treating the fully grown colonospheres on day 14. AM404 treatment showed distinct morphological alteration in colonospheres, which could be related to cell polarity, cell-cell attachment, EMT, resulted by differentiation induction; however, the number of colonospheres remained the same after the treatment (Figure 2C). Characterization of the inhibition pathway involved in the mechanism of action of AM404 on CSC-like properties and the sensitivity to chemotherapeutics, as evidenced in the colonospheres, was initially performed by gene expression analysis from mRNA isolated from colonospheres of roughly the same size using qRT-PCR analysis. Notably, a significant reduction in expression level was observed for *CD44*, *NANOG*, *LGR5*, *OCT4* and *BMI-1* stem cell markers, *CXCR4* and *c-JUN* oncogenes, whereas, *KRT20* and *CDX2* differentiation markers and *FBXW7* tumour suppressor genes were significantly increased upon AM404 treatment in the colonospheres (Figure 2D–F). Furthermore, immunofluorescence assay on AM404 treated colonospheres with well-established differentiation marker MUC2 and stemness CD44 also revealed high expression pattern with MUC2 (Figure 2G,H). Thus, several established stem cell markers, CRC prognostic factors, and differentiation markers have also revealed the potential of AM404 in targeting stem-like cells.

**Figure 3:**
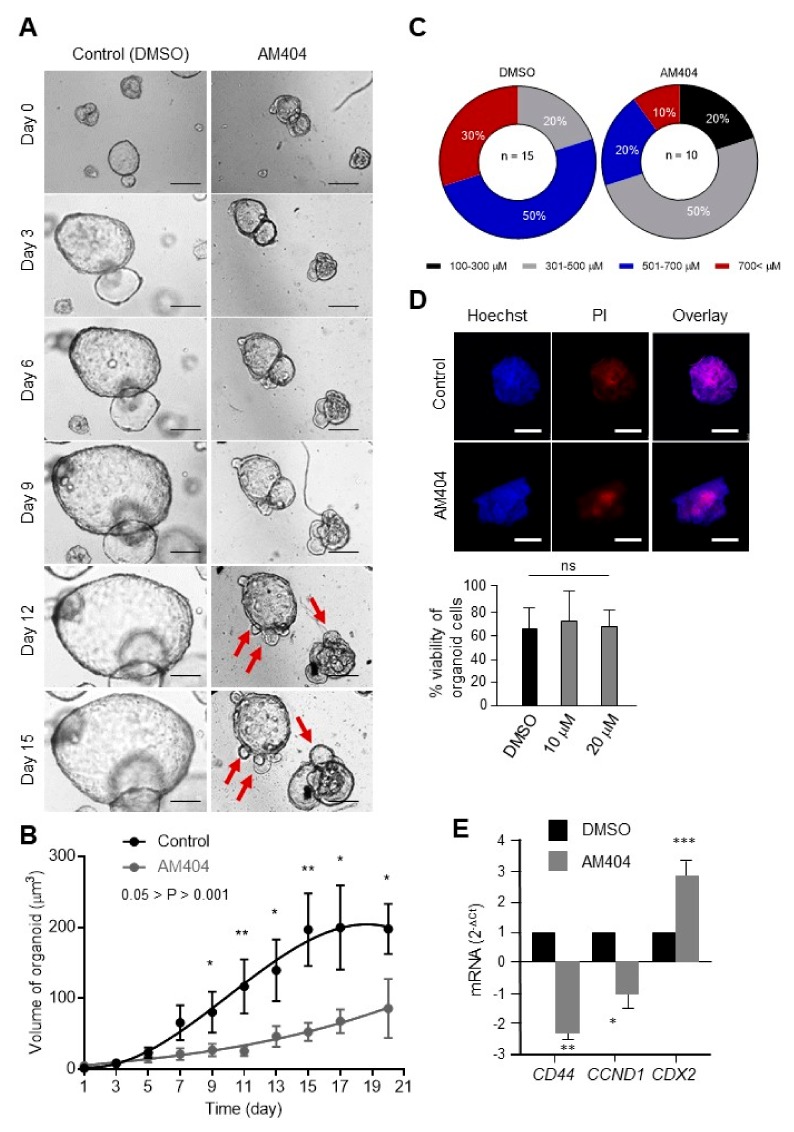
AM404 treatment altered CRC patients-derived organoids growth and morphology patterns. (**A**) Representative images of tumour organoids treated with AM404 for a period of 2 weeks. Tumour organoids were cultured and allowed to grow for 5-6 days prior to the treatment. AM404 was added to its IC_50_. Both AM404 and control-treated groups were maintained with changing of medium every other day. Morphological alteration and variation in growth were observed throughout the treatment period. Images were taken using Leica microscope. Scale bar: 75 µm. (**B**) AM404 treatment causes growth impairment in tumour organoid. Volumes of organoids were measured every 2 days. AM404 treatment shows significant inhibition to the tumour growth as compared to control-treated group. Error bars represent mean ± SEM. (*n* = 15; control, *n* = 10, AM404). 0.05 > *p* > 0.001. (**C**) Number of organoids in different size groups at the end of the treatment. Numbers in each size group are expressed as percentage of the total. In treated group, only 10% of organoids were measured as more than 700 µM, as compared to the 30% of total organoids in control group. (**D**) Hoechst/PI staining on AM404 treated organoids. The similar pattern in PI staining in both AM404 and control-treated group indicates no changes in cell death upon AM404 treatment. Scale bar: 25 µM. (**E**) qRT-PCR analysis of AM404 treated compared with control untreated patients derived organoids for CD44, cyclin-D1 and CDX2. Student’s *t*-test was performed for the statistical analysis. *, *p* ≤ 0.05; **, *p* ≤ 0.01; ***, *p* ≤ 0.001.

As a first step towards mimicking the patient tumour tissues, we used CRC patients’ derived organoids [42,43], to study specific cell-type response to drugs [44]. Organoids were cultured and allowed to start budding for 5–6 days prior to treating them with AM404. The 2 weeks treatment period was chosen based on the majority of control-treated organoids grew >700 µm and covered the limited space within the wells after a total period of 3 weeks. When compared to control-treated tumour organoids, AM404 appeared to induce distinct morphological changes such as branching formation (Figure S3), which may indicate of AM404-induced differentiation (Figure 3A, arrowheads, and Figure S3). The volume of organoids was significantly smaller in the AM404-treated group (197 µm^3^ vs. 86 µm^3^; 0.05 > *p* > 0.001). (Figure 3B). At the end of the treatment, only 10% of organoids were measured to be as more than 700 µM, as compared to the 30% of total organoids in control group. Similarly, in reference to all the organoids being larger than 300 µM in control group, 20% organoids were still within the range of 100–300 µM in AM404 treated group, on day 14 of the treatment (Figure 3C). This result shows a high population of AM404-treated organoids being in smaller diameter range, suggesting its inhibitory effect on organoid growth. However, cell dead/alive status identified by PI/Hoechst staining. AM404 treatment showed slight/no reduction in PI staining followed by 6 days treatment period in tumour organoids (Figure 3D), while differentiation markers *CDX2* expression were significantly increased (Figure 3E). In addition, after AM404 withdraw on day 14 of the treatment, signs and symptoms of the differentiation, only some of the treated organoids were reversed. This result suggests minimal/no changes in cell death caused by AM404 on tumour organoids. Our results are consistent with previously reported evidence that AM404 is well tolerated on mammalian cells and in animal models [22,23]. Taken together, these results may indicate that AM404 induces differentiation and thereby affects CRC-SCs.

### 2.3. Ex Vivo Treatment of CRC Patient Biopsies Evaluates AM404 Response As an Anticancer Drug

Our results from monolayer cells, colonospheres, and patient-derived organoids have shown that AM404 impedes the growth and can induce morphology change while reducing the stem-like properties. Further studies towards mimicking the clinical response of the tumour environment, an ex vivo platform capturing tumour heterogeneity was developed. Ex vivo explants have previously been reported to be more viable in the short-term culture method [45,46]. Fresh tumour tissues were obtained from CRC patients following their surgery and processed immediately for culturing explants. To validate AM404’s response on stemness, differentiation and proliferation deliberated in tumour tissues, we have selected 15–17 h for the explants to recover after initial generation, based on published data [45,46]. Following analyses of AM404 responses were also performed for 24 h, at its IC_50_ (Figure 1E)_._

**Figure 4:**
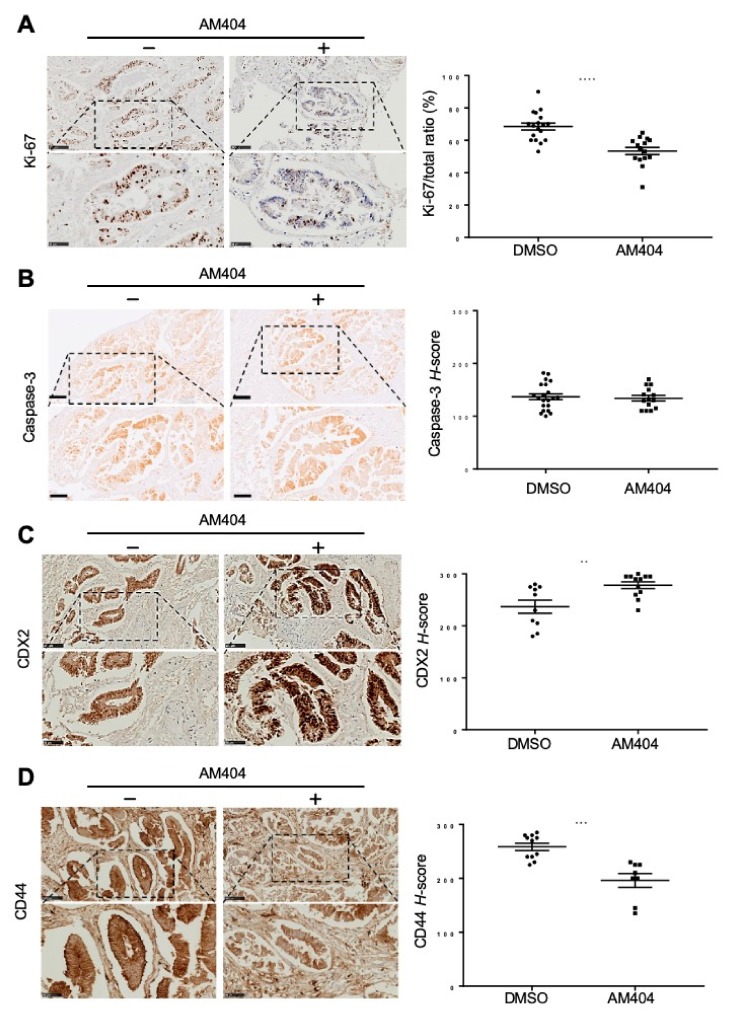
AM404 treatment impacts on cancer cell proliferation, stemness and differentiation in the patients-derived CRC tissues explants (7 patients). (**A**–**D**) Proliferation was assessed by quantitating Ki-67 (A) IHC staining, cell survival by caspase-3 (**B**), whereas differentiation and stemness levels were assessed by CDX2 (**C**) and CD44 (**D**) staining quantification respectively. IHC stains were counterstained with hematoxylin and eosin. Representative images (top panel) shows the images at 20× magnification with a scale bar of 100 μm. Bottom panel shows the selected parts in higher 40× magnification with scale bar 50 μm in control (DMSO) and AM404-treated groups. Each dot (Right panels) represents an image used for quantification. Student’s *t*-test was performed for the statistical analysis. Control treated groups represents the level of intrinsic proliferation, stemness and differentiation level in tumours. For the Ki-67 staining, Ki-67 + ve cells were counted and expressed as a percentage of the total cells. In case of CD44 and CDX2 and Caspase-3, H-scores were counted as a measure for the quantification. AM404 treatment showed significant reduction in proliferation and stemness levels, and significant increase in the differentiation level in tumour explants.

IHC analysis of AM404 treated explants showed a trend of decreasing cell proliferation and stemness with increasing differentiation marker (Figure 4). This was found to be consistent for tumour explants generated from all the CRC patients, with 5–6 images analysed per tumour sample. Overall, 24-h treatment with AM404 significantly reduced Ki-67 staining and the proliferation level in tumour explants by 20% (Figure 4A, right panel). However, Caspase-3 staining showed, not a significant number of cells will necessarily die in response to AM404 (Figure 4B). Therefore, to associate AM404 with cellular differentiation and stem-like activity, we utilized CDX2 and CD44v6 as markers for differentiation and ‘stemness’ in tumour explants (Figure 4C,D). 

Overexpression of CDX2 has been shown previously to induce differentiation as well as to inhibit proliferation and is therefore, frequently downregulated during tumorigenesis [47]. We used the H-score system to quantify CDX2 expression in both control-treated and AM404 treated groups (Figure 4C, left panel). Our result showed a significant increase in CDX2 expression in tumour explants treated with AM404 (Figure 4C, right panel). CD44v6 is a multifunctional transmembrane glycoprotein and it has long been used as a marker of colorectal cancer stem cells, and is associated with cell adhesion, growth, differentiation, migration and tumour progression. CD44v6 positive cells have been reported to have the characteristics of stem cells and have a higher level of proliferation and invasion than CD44v6 negative cells [48,49]. 

In line with the proliferation pattern, CD44v6 expression was also reduced by AM404 treatment on the tissue explants (Figure 4D, right panel). Thus, treating these tumour explants with AM404 showed a reduction in proliferation and stemness while also increasing the level of differentiation characteristics.

### 2.4. FBXL5 Attenuates AM404-Induced Anticancer Activity

**Figure 5:**
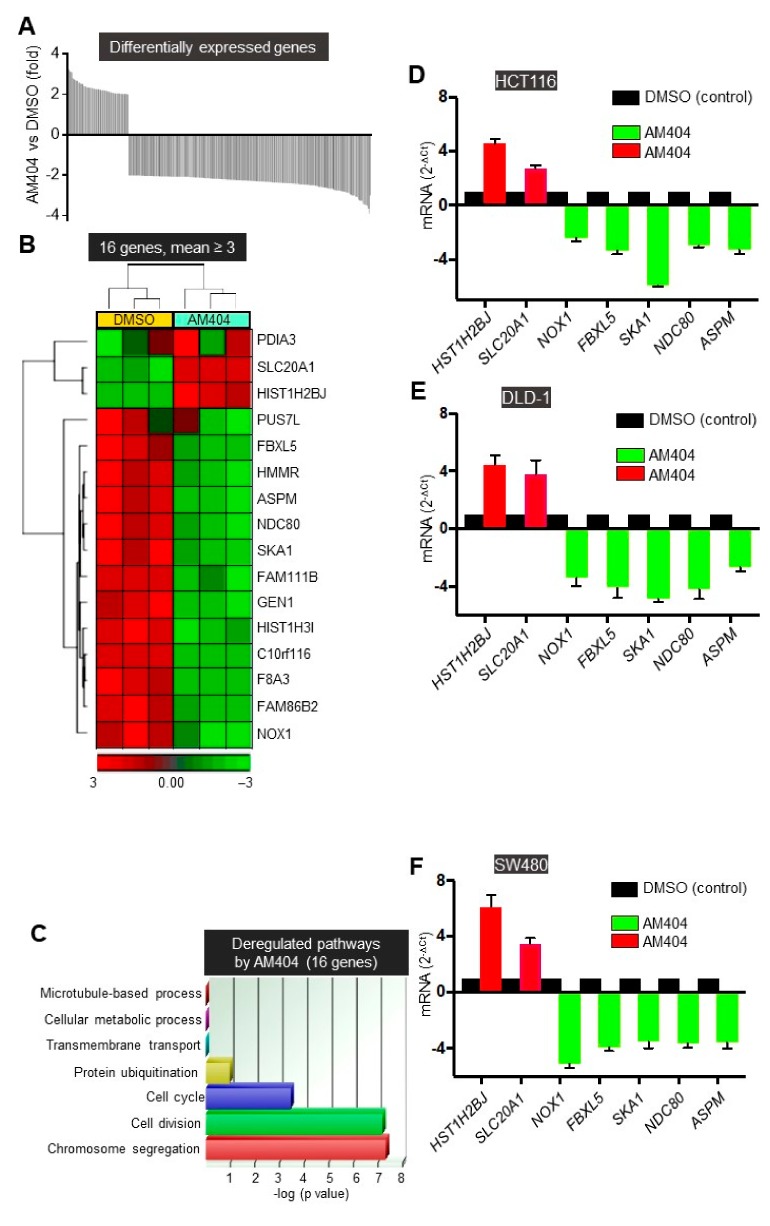
AM404 treatment altered genes expression profile of DLD-1 colonospheres. (**A**) Differentially expressed genes on colonospheres treated with AM404. Each bar represents one gene that has a *p*-value of 0.001 or less and is 2 or higher fold increased or decreased on colonospheres following the drug treatments. (**B**) Heat-map representation of unsupervised clustering of the 16 differentially expressed genes (mean ≥3-fold change) by AM404 in colonospheres. Each row represents a gene. Each column represents a sample: yellow, control (DMSO treated) and blue, AM404 treated colonospheres. Colour code within the graph represents log2 of the fold change of expression: green, downregulated; red, upregulated. Horizontal and vertical clusters were created based in Euclidean distance. (**C**) Gene ontology biological processes revealing the top biological processes affected by the sixteen genes represented in B. Functional annotation clustering with default settings was used; medium stringency and Benjamini–Hochberg correction was applied. Only the enriched GO terms with FDR < 0.05 were selected and displayed in the bar chart. The full list of differentially expressed genes can be found in Tables S2 and S3 and their GO biological processes enrichment in Figures S3 and S4. (**D**–**F**) mRNA expression levels of seven genes with differential expression including *FBXL5* by AM404 obtained from the microarray analysis were confirmed by RT-qPCR analysis in colonospheres derived from HCT116 (**D**), DLD-1 (**E**) and SW480 (**F**) cells. Student’s *t*-test was performed for the statistical analysis. Error bars represent SEM.

To identify the targets associated with AM404′s mechanism on CRC, the transcriptome of DLD-1-colonospheres treated with AM404 and controls was compared. Microarray analysis emphasizing on 2 or more-fold changes (*p* < 0.001) revealed 323 differentially expressed genes (Figure 5A, Figure S4 and Table S3). As predicted by the status of colonospheres, the gene ontology (GO) pathways for 75 genes with 2.5 or more-fold changed were mainly associated with cell cycle, DNA damage, and protein ubiquitination/ degradation signalling (Figure S5A,B). Among these top biological processes, and 16 genes with changes of 3-fold or greater (Figure 5B,C), protein ubiquitination was one of the major gene expression regulatory groups. In general, the specificity of proteolysis for any particular substrate is determined by its association with a specific E3-receptor subunit. F-box proteins are the substrate-recognition components of the Skp1-Cul1-F-box-protein (SCF) E3-ubiquitin ligases. Accordingly, F-box proteins can function as oncoproteins when overexpressed (if their substrates are tumour suppressors) or as tumour suppressors (if their substrates are oncoproteins). For example, we have extensively studied FBXW7, a commonly mutated tumour suppressor gene in human tumours including 10–15% of CRC, which, we found to be significantly increased upon AM404 treatment (Figure 2D) [34,39,50]. However, characterization of many other F-box proteins is required for their roles in cancer, which could be a key breakthrough for cancer therapy and offer a potential new biomarker(s) for early detection of epithelial tumour progression including CRC. Therefore, we have selected *FBXL5* gene, which showed over three folds decrease upon AM404 treatment (Figure 5B–F), functioned as an oncogene in the progression of colon cancer through regulating PTEN/PI3K/AKT signalling [24] and HIF-1α transcriptional activity [26].

FBXL5 (F-box and leucine-rich repeat protein 5), also known as FBL4 and FBL5, is a member of the F-box protein family, characterized by an F-box motif consisting of 40 amino acids [24,26,51]. It is predominantly an iron and oxygen-regulated SCF-type E3 ubiquitin ligase containing an N-terminal hemerythrin-like domain, α-helix-rich structure [51,52]. Therefore, we initially sought to examine whether the expression of *FBXL5* was altered in patients’ colorectal cancers.

**Figure 6:**
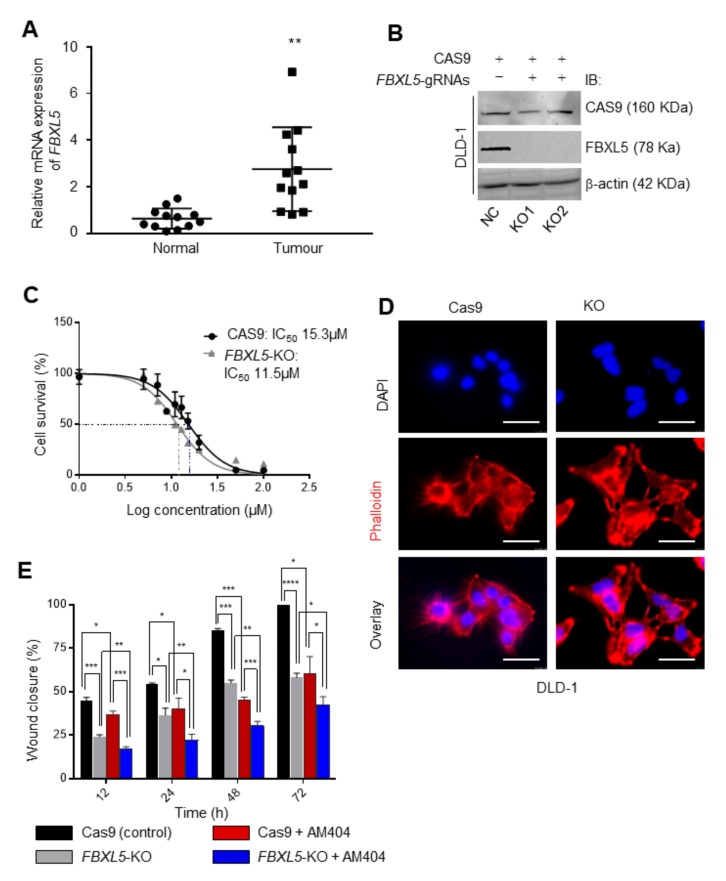
AM404 association with FBXL5 in CRC. (**A**) qRT-PCR analysis of *FBXL5* mRNA expression in a cohort of twenty-two; normal adjacent and tumour tissues from patients with CRC in Nottingham, UK, normalized to Hypoxanthine-guanine phosphoribosyltransferase (HPRT). Student’s *t*-test was performed for the statistical analysis. Data are mean ± SEM (*n* = 3; **, *p* ≤ 0.01). Experiments were performed in triplicate for each sample and repeated on two independent occasions. (**B**) Western blot analysis of FBXL5 expression in DLD-Cas9 (control) and DLD-Cas9:FBXL5-gRNAs (CRISPR-knockout) cell lines (KO1 and KO2). β-actin was used as loading control (Figure S7). (**C**) IC_50_ of AM404 on *FBXL5*-KO cell lines. FBXL5-KO cells are found to be more sensitive to AM404 with an IC_50_ of 11.5 µM. (**D**) FBXL5 modulates cell adhesion and morphology. These cells were stained with Phalloidin and visualized under fluorescent microscope. Scale bars, 50 μm. (**E**) AM404 significantly inhibited migration in DLD-1 and FBOXL5-KO-DLD-1 cells in vitro. Migration of DLD-1 and FBOXL5-KO-DLD-1 cells was performed with scratch wound healing assay. Starved cells were scratch-wounded and wound width was measured to determine the healed distance (please see Figure S6B). Significant reduction in cell migration was observed for both cell lines. Bars are expressed as mean ± SEM. (*n* = 3). *, *p* < 0.05; **, *p* < 0.01; ***, *p* < 0.001; ****, *p* < 0.0001.

These results indicated a lower expression of *FBXL5* mRNA in normal/healthy tissues adjacent to the tumours (Figure 6A). Notably, Yao et al., showed post-surgical patients with high expression of *FBXL5* had shorter overall survival than patients with low FBXL5 expression [24]. Based on our data suggesting AM404’s effect in CSC-like activity (Figure 2), and recent studies indicating FBXL5 regulating CRC metastasis [23,25,26], we then wanted to elucidate whether AM404 exerted its noted effects via FBXL5 in CRC cells [24,26,27]. We have therefore generated CRISPR-Cas9 mediated *FBXL5*-knockout in DLD-1 cell line (Figure 6B and Figure S6). The cytotoxicity assay of the *FBXL5*-knockout cell line further confirmed that loss of FBXL5 induced sensitivity of cells (11.5 µM vs. 15.3 µM; 0.05 > *p* > 0.001) to AM404 than that of the control-treated cells (Figure 6C). In addition, knockout of *FBXL5* caused an inhibition of colony formation efficiency in DLD-1 cells (KO1 & KO2) (Figure S6A). Our microscopic study for Phalloidin stained DLD cells showed that *FBXL5*-knockout cells (KO) displayed branched, flat, and elongated shape with prominent actin fibers (Figure 6D). Furthermore, we synchronised cells by serum starvation, and performed scratch wound healing assays [53]. The result showed that AM404 significantly reduces the migration of DLD-1 cells (Figure S6B and Figure 6E, Black vs. Red columns). Consistent with the cell morphology observation, the wound-closure of *FBXL5*-knockout cells versus control cells expressing Cas9 is significantly reduced (Figure S6B and Figure 6E, Black vs. Gray columns). AM404, being sensitive to *FBXL5*-knockout cells, showed to mimic this effect and caused further additive effect in inhibiting cell migration (Figure S6B and Figure 6E, Red vs. Blue columns). Furthermore, in between two treatment groups, AM404 treatment caused significant reduction in cell migration in *FBXL5*-knockout cells than that of WT DLD-1 cells (Figure 6E). This finding postulates FBXL5 as a potential target via which AM404 exerts its effects on CRC cells migration.

## 3. Discussion

In summary, the 3D models used throughout the study provides means of reproducible, rapid, low cost and patient relevant platform not only for drug screening, but also for the preclinical evaluation of novel anticancer agents. Based on this platform, we have identified the anti-bacterial AM404 as a potential candidate to target CRC cells via supressing the oncogenic E3 ligase FBXL5. We speculate that AM404 modulated FBXL5 expression might reduce polarized epithelial cells to inhibit migration to distant sites.

Infection and chronic inflammation are major causes of cancer. Our understanding of the molecular pathways and links between inflammation and cancer is continuously improving. AM404 is currently being studied for its antibacterial and anti-inflammatory effects notably for reducing the production of IL-1β, IL-6 as well as decreasing oxidative stress and for their association with circulating tumour necrosis factor (TNF)-α [19]. Previously, AM0404 has been reported to inhibit NF-κB and NFAT activation on neuroblastoma and glioma cells. NF-κB is one of the key transcription factors in cancer associated inflammation transition process, regulated via TNF and various cytokines including IL-6 [54,55]. In neuroblastoma cells, AM404 inhibited the NF-κB activation by targeting IKKβ phosphorylation and activation 57 In addition, AM404 also impaired COX-2 expression, PGE2 release, migration and invasion in a cell specific manner [56,57]. Inhibition of COX-2 has an anti-tumorigenic effect in cancers that occurs due to prolonged chronic inflammation. Several pathways including Wnt–β catenin, PIK3CA/AKT/PTEN, and NF-κB, have been postulated as targets for NSAIDs [54]. FBXL5 has previously been interpreted as oncogene whilst its association in iron regulation is required for HSC self-renewal [52]. In CRC progression, FBXL5 has shown to induce cell proliferation, growth, tumorigenesis and inhibit cell apoptosis by modulating PTEN/PI3K/AKT signalling and its overexpression resulted in high tumour formation ability [24,27]. Furthermore, they have also been reported to negatively regulate several EMT inducers, such as Notch, c-Myc and mTOR, particularly in gastric and cervical cancer [24,51]. We showed that AM404 associated cell homeostasis caused reduction of CRC ‘stemness’ features, including cell proliferation, migration, tumour growth, morphology, and induction of CRC differentiation. It has been markedly reported that, silencing FBXL5 showed decrease in metastasis with significant increase in expression of E-cadherin at posttranscriptional level [27]. In line with this, we showed that AM404 significantly reduced the expression level of N-cadherin and Vimentin (Figure 2E), and by mimicking the effect of *FBXL5* deletion, it also significantly prevented the invasion in CRC cells (Figure S5B and Figure 6E). When used in FBXL5-KO-cells, AM404 showed sensitivity and further additive effect in preventing cells invasion suggesting AM404 as a new compound to target FBXL5 in blocking CRC cell migration. We currently do not have an in vivo intestinal/colon model that implicit FBXL5 as a potential therapeutic target for cancer stem cells, although an essential role of FBXL5-mediated cellular iron homeostasis in the maintenance of hematopoietic stem cells has already been reported [52].

This study demonstrates the application of colonospheres, organoids and explant models to screen new compounds and helps improving our understanding of the inflammatory mediators involved in CRC. This study thereby also helps reducing, and potentially replacing animal models and may provide novel preventive, diagnostic and therapeutic strategies.

One of the major hallmarks of cancer highly focuses on inflammation specifically for cancer progression, development and proliferation. Due to this correlation, preclinical focuses highlighted several antibacterial, anti-inflammatory drugs for repurposing in cancer treatment. In the study of Sharma et al., [19] AM404 was detectable in plasma of only eight of the 26 plasma samples, with three being above 5 nmol/L, which warranted an unidentified mechanism in some individuals through which AM404 leaks out of the brain and into the blood. In most cases, otherwise, the plasma concentration was below the detectable range. However, when we used the measured IC_50_ in colonospheres, organoids or tissue explants systems that recapitulate patient response, this concentration showed induction of differentiation rather than cell toxicity or cell death as evident in Figure 3 and Figure 4. The heterogeneous cell population could explain why the cells may respond differently to treatment in these systems [58]. In addition, the concentration at which different drugs exert their pharmacological effects varies largely on the dynamics of the drugs and different cell types. Many drugs are used at concentrations of nM–mM range, with concentrations >10 μM. For example, BBI608 with 30 μM, 20 μM ECGC, 10 mM metformin, or vismodegib 10 µM [59,60,61,62]. Therefore, we presume this concentration may not be too toxic for future clinical use.

Based on our results, we conclude that AM404 as a new compound and FBXL5 as an associated key target gene with high therapeutic pharmacological potential could be used against human colorectal cancer and other infectious diseases.

## 4. Materials and Methods

### 4.1. Human Tissues

Tumour and adjacent healthy tissue samples of 17 CRC patients were collected [43] from Nottingham Health Sciences Biobank (NHSB), Queens Medical Centre, University of Nottingham. Ethical approval and research and development approval including written informed consent were obtained by Nottingham Health Science Biobank (NHSB), Histopathology Department, School of Medicine, University of Nottingham, Nottingham NG7 2UH. We collected samples from Biobank via access committee of NHSB approval number: ACP000098 A Nateri CRC. Tissue samples were used for culturing patient-relevant 3D organoid and ex vivo tissue explant models [45,46]. All procedures were conducted following the Declaration of Helsinki and local ethics committee approval. Tissue samples were collected only from patients who provided written informed consent.

### 4.2. Ex Vivo Explant Culture of CRC Tissues

Explant tumour models [45,46] were cultured from patients’ tissues obtained as outlined above. Primary tumour tissue samples were cut in 2–4 mm segments and were maintained in complete organoid medium supplemented with Noggin 12–15 h or overnight. Explant samples were treated with the drug for 24 h. Tissues were fixed overnight in 10% neutral-buffered formalin (NBF), paraffin embedded, and sectioned at 4-mm thickness for hemotoxylin and eosin (H&E) staining or immunohistochemistry (IHC).

### 4.3. Organoid Culture

CRC patient-derived organoids were cultured as previously established and characterized in our lab [42,43]. Colonic crypts were made to release from intestinal epithelium and were re-suspended in Matrigel in the presence of complete organoid medium [43]. Once the organoids started to grow (usually day 4–5), they were treated with drug and/or complete organoid medium as vehicle control for their morphology, growth evaluation and live/dead staining. Considering the 3D enteroid structures with crypt like projections, volume of organoids was measured by taking 3–4 separate diameters for each organoid. Half of the average of this diameter was considered as radius (r), organoid volume was measured using the following formula: 𝑉 = 4/3𝜋𝑟^3^. Re-treatment and/or organoid medium replacement was carried out every two days, for the whole experimental period. Microscopy was performed using a DMI3000 B fluorescence microscope (Leica Biosystem, Milton Keynes, UK) at ×10 and ×40 magnification for recording organoid growth and/or live/dead staining.

### 4.4. Organoids Live/Dead Staining

Live organoids were stained with Hoechst 33342, a blue-fluorescence dye to stain all cells and propidium iodide (PI, Sigma Aldrich, Dorset, UK), a red-fluorescence dye to stain dead cells, according to double Hoechst 33342/PI stain apoptosis detection kit (GenScript, Leiden, Netherlands). Stained organoids were carefully collected and fixed, in order to distinguish live/dead staining with the Leica DMI3000 B fluorescence microscope.

### 4.5. Immunohistochemistry (IHC)

Fixed tissues were processed by a Leica TP1020 semi-enclosed benchtop tissue processor via automatic passages from ethanol (70%, 90% and 100%) to methanol, xylene and, lastly, paraffin. Samples were embedded in paraffin blocks to be cut in 4 µm-thick sections with a microtome and placed onto the glass slides for IHC analysis. Immunohistochemical analysis was carried out with and without (antibody control) Ki-67 (Dako, Stockport, UK), active caspase-3, CDX2, CD44 (Cell Signaling, London, UK), and FBXL5 (Abcam, Cambridge, UK) antibodies followed by incubation with secondary antibodies and detection reagents. A section from a CRC tissue known to express the protein of interest was also used as positive control [33]. Slides were scanned at 20× magnification. Images were analysed using NanoZoomer Digital Pathology software (Hamamatsu Ltd., Welwyn Garden City, UK). Immunostaining was evaluated by H-score method to calculate the sum and intensity of positively stained tumour cells. The H-score is ranged from 0 to 300, using the formula: (1 × % weakly stained nuclei) + (2 × % moderately stained nuclei)  +  (3 × % strongly stained nuclei) [63].

### 4.6. Cell Culture and Colonosphere Formation Assay

Human colorectal carcinoma HCT116, DLD-1, and SW480 cell lines were used throughout the study. These were purchased from ATCC and were further characterised in our laboratory [33,34,64]. Cells were routinely tested and approved mycoplasma-free. All cells were propagated in complete medium and used for experiments within 5 passages from thawing. 3D colonospheres were cultured for 13–14 days as previously established [15,16]. Well-defined colonospheres were then treated with drug compound for 72 h for further employment into NIH clinical collection screening, colonosphere formation and morphology evaluation, immunofluorescence assay, RNA extraction for qRT-PCR and gene array.

### 4.7. Screening of NIH Clinical Collection Using 3D Colonosphere

As a representation of CRC-SC [16], colonospheres obtained from the above CRC cell lines were employed into a fluorescence-based screening of US National Institute of Health (NIH, Evotec, South San Francisco, CA, US) clinical library consisting of 707 small molecule inhibitors. The fluorescent Rosamine dye CDy1- (Active Motif, La Hulpe, Belgium) based screening in 96 well-plates was used to examine the effect drugs in the differentiation/stemness activities of colonospheres [17,18]. Fluorescence intensities were measured using a CLARIOstar microplate reader (BMG LABTECH, Aylesbury, UK) with optic setting for excitation and emission as 544–10 and 577–10 nm respectively. Based on this platform, throughout the screening, fluorescence intensity of vehicle (DMSO) treated colonospheres was used as control. All treated colonosphere intensities were expressed as a percentage of the control as an indication of induced or reduced stem-like activity.

### 4.8. Cytotoxicity Assay

Same passage number of HCT116, DLD-1 and SW480 cells were seeded in 96-well plate and were allowed to grow. Subsequently, cells were serum-starved for 18  h and then were treated with a drug by itself or with 5-FU and Oxaliplatin (Tocris, Abingdon, UK) for 72 h. Sulforhodamine-B colorimetric assay (230162, Sigma Aldrich, Gillingham, UK) was performed as previously described [33].

### 4.9. Clonogenic Assay

DLD-1 cells were seeded in 6-well plates (200 cells per well). Cells were allowed to form into colonies and were treated with AM404 on day 7. Colonies were re-treated or replaced with fresh medium every three days throughout day 14. They were then fixed with 4% paraformaldehyde and stained with 0.01% crystal violet (Sigma Aldrich, Gillingham, UK), before the manual counting and colony size measurement.

### 4.10. Cell Migration and Wound Healing Assays

Wound healing assay was conducted on DLD-1 cells to investigate AM404’s effect on migration [53]. Cells were cultured in a monolayer confluent manner. In order to suppress cell proliferation and avoid interference with the migration measurement, they were serum starved for 18 h. A wound was stimulated using pipette tip, creating gap in the confluent monolayer cells and removed of any mechanical debris by subsequent washes. Images were taken for the time point of 0 h, using a phase-contrast microscope. Cells were treated with the drug and/or medium immediately and returned to the incubator. Image acquisition integrity was assured by several reference points close to scratch. Images were taken periodically at time points of 12 h, 24 h, 48 h and 72 h following the abovementioned approach. The distances between the scratch sides (μm) were measured and compared between 0 h and 72 h.

### 4.11. Immunofluorescence and Western Blotting

Immunofluorescence and Western blotting analyses were conducted to study the expression pattern and distribution of a protein within cells as previously described [15].

### 4.12. Knockout of FBXL5 Using the CRISPR/Cas9 System

Two copies of 19- and 20-bp guide sequence targeting DNA within the first and eleventh exon of FBXL5, with high-specificity protospacer adjacent motif (PAM, Sanger, Cambridge, UK) target sites was cloned in LV04 Sanger Lentiviral CRISPR vector (Sigma) respectively. DLD-1 and SW480 stably expressing Cas9 cells were transduced with LV40-FBXL5 gRNAs. Single transduced cells were isolated by puromycin selection and individual clones extended and screened by immunoblotting with anti-FBXL5 antibody (Abcam). Genomic DNA was isolated from individual edited clones, and PCR amplified exons products were sequenced to confirm homogeneous representation in the edited cells.

### 4.13. RNA, Transcriptomic, qRT-PCR Assay

Total RNA was isolated from CRC cells, colonospheres and tissues with TRIzol reagent (Sigma Aldrich) and RNeasy Mini Kit (QIAGEN) following the manufacturer’s protocol. The quality and integrity of the total RNA were evaluated on the Agilent-2100 Bioanalyzer system (Agilent, Stockport, UK). Only samples surpassing the minimal quality threshold (RIN > 8.0) were used in the subsequent transcriptomic assessment. cDNA was prepared from 200 ng of RNA as per the GeneChipTM WT-PLUS Reagents (Thermo Fisher Scientific/Affymetrix, Winsford, UK), and followed by in vitro transcription to produce cRNA, end-labelled and hybridized for 16 h at 45 C to GeneChip™ Human Gene 2.1 ST Arrays (Thermo Fisher Scientific/ Affymetrix, Winsford, UK). All steps were performed by a GeneAtlas™ Personal Microarray system (Thermo Fisher Scientific/ Affymetrix, Winsford, UK) according to manufacturer’s instructions at the Nottingham Arabidopsis Stock Centre (NASC, School of Biosciences, and University of Nottingham). Differentially expressed genes were considered significant if *p*-value with FDR ≤ 0.05 and fold-change of >2 or <−2.

Transcriptomic data were then processed by a standardized sequence of analyses (gene ontology (GO) enrichment) using Ingenuity Pathway Analysis. For qPCR assays, cDNAs were generated by using PrimeScript RT Reagent Kit (Perfect Real Time) (Takara-Clontech Laboratories, Saint-Germain-en-Laye, France) and cDNA samples were then amplified using LightCycler 480 SYBR Green I Master Mix (Roche, Welwyn Garden City, UK) and LightCycler 480 II instrument (Roche). Results were normalized to those obtained with β-actin, and all assays were performed in triplicate. Details of primers used are shown in Table S1.

### 4.14. Data Analysis and Statistics

GraphPad Prism 7 (GraphPad Software, San Diego, CA, USA) and Microsoft Office Excel (Microsoft, Redmond, WA, USA) were used to generate graphs and carry out statistical analysis. Fiji (ImageJ) software (ImageJ 1.51j8, NIH, Bethesda, MD, USA) was used to analyse images. Gene expression data were analysed using Partek Genomics Suite 7.0 (Partek Incorporated, St. Louis, MO, USA). Data are reported as means ± SEM using the Student *t* test and the Mann–Whitney *U* test, as appropriate and for all analyses, *p* < 0.05 was considered statistically significant. * *p* < 0.05; ** *p* < 0.01; *** *p* < 0.001 values are shown.

## 5. Conclusions

Our data demonstrate a new molecular mechanism, by which an uncharacterised antibacterial AM404 drug altering the oncogenic activity of FBXL5 receptor subunit of E3-ligase, to alter differentiation, migration and drug-resistant of CRC cells. Needless to say, that, the connection between inflammation and tumorigenesis involved at different stages during pathogenesis in all malignancies, and therefore these cancer-related cellular processes alterations through AM404 may offer possibilities for the anticancer potential of AM404 targeting the FBXL5-E3 ligase signalling in different types of cancers.

## Supplementary Materials

The following are available online at https://www.mdpi.com/2072-6694/12/1/106/s1. Table S1: List of primers and their sequences used for qRT-PCR assay, Table S2: List of drugs selected during different stages of the screening, Table S3: List of differentially expressed genes upon AM404 treatment, Figure S1: Optimisation of screening methodology using CDy1, Figure S2: Isobologram analysis shows the combined effect, Figure S3: AM404 treatment presents enteroid-like structures and induces differentiation in organoids. Percentage of enterosphere, cyst, enteroids and dead cells at day 3, 7 and 15 days of treatment, Figure S4: Principal components analysis (PCA) of microarray data between DMSO (control, red) and AM404 treated (blue) cells transcriptomes, Figure S5: Gene ontology (GO) pathway for 75 differentially expressed genes (2.5 or more-fold change), Figure S6: Clonogenic assay of AM404 on *FBXL5* KO DLD-1 cells, Figure S7: Whole Western Blot image and intensity ratio.
