# Supplementary material for: Repurposing Antibacterial AM404 As a Potential Anticancer Drug for Targeting Colorectal Cancer Stem-Like Cells
Source: Cancers (Basel). 2019 Dec 31;12(1):106. doi: 10.3390/cancers12010106 (PMC7017077; doi:10.3390/cancers12010106)
Supplement: Supplementary file 1 [file cancers-12-00106-s001.pdf]

# Supplementary material: Repurposing Antibacterial AM404 As a Potential Anticancer Drug for Targeting Colorectal Cancer Stem-Like Cells

**Mehreen Ahmed, Nicholas Jinks, Roya Babaei-Jadidi, Hossein Kashfi, Marcos Castellanos-Uribe, Sean T. May, Abhik Mukherjee and Abdolrahman S. Nateri**

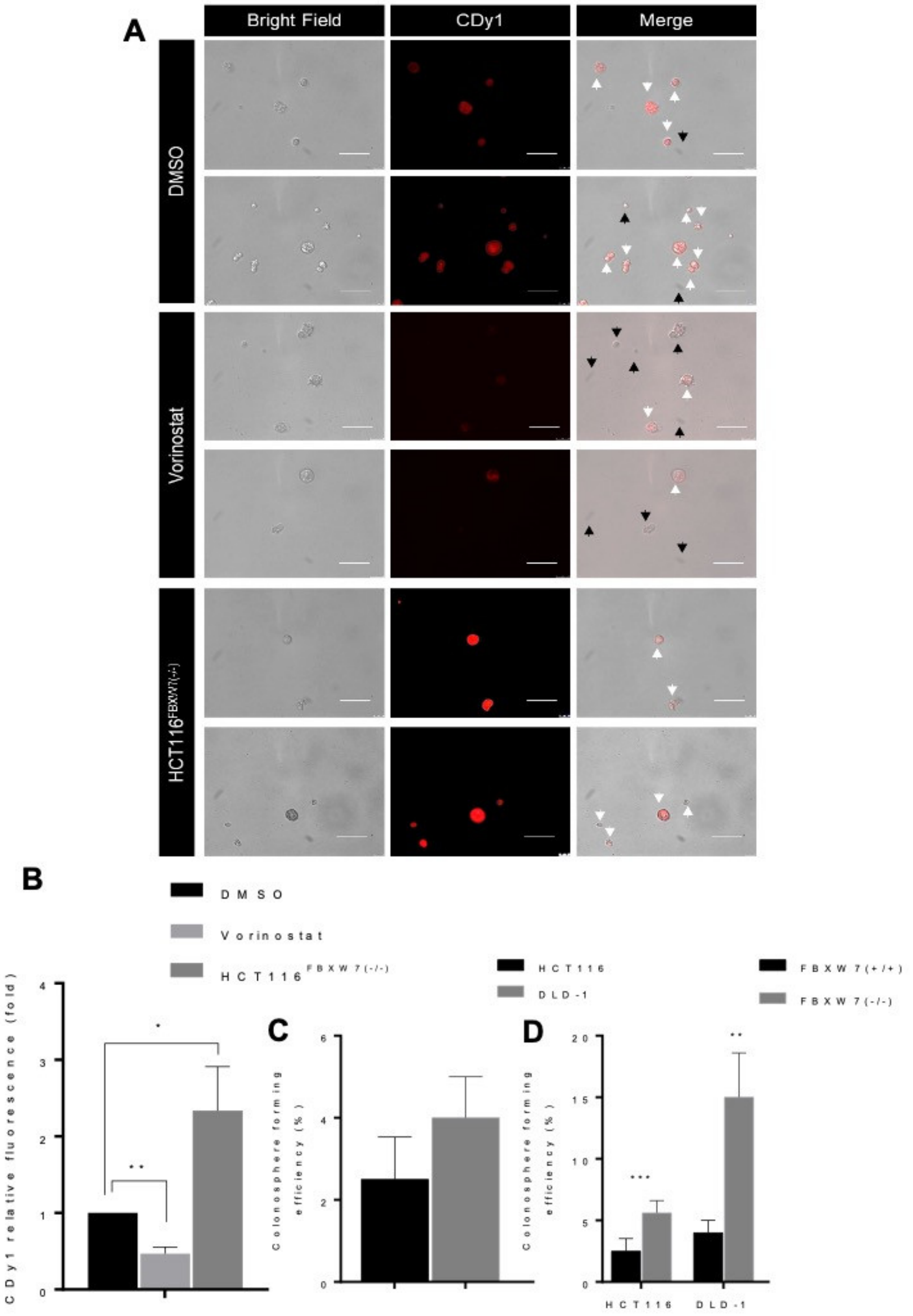


**Figure 1. A**) Vorinostat (SAHA) induces differentiation and FBXW7 deletion indicates attainment of more stemness activity in HCT116 derived colonospheres. CDy1 dye marks the stem-like activity of formed colonospheres. Black arrows show the colonospheres that were not stained with CDy1, indicating loss of their stem-like activity. Whereas, white arrows show the colonospheres with stem-like characteristics. Scale bars: 25 μm. **B**) CDy1 confers reduced fluorescence intensity in Vorinostat

treated colonospheres, possibly due to the drug induced differentiation and greater intensity in HCT116-FBXW7(−/−) cell derived colonospheres indicating higher tumorigenic properties as observed by CDy1 staining. Bars represent mean ± SD, $n = 15$; * $p < 0.05$, ** $p < 0.01$, using Student's *t* test. **C**) Quantification of the colonosphere-forming ability of the HCT116 and DLD-1 cell lines. **D**) Quantification of the colonosphere-forming ability of the HCT116 cells ± FBXW7 (left) and DLD1 cells ± FBXW7 (right). Bars represent the mean of three different experiments ± SEM; ** $p < 0.01$, *** $p < 0.001$, using Student's *t* test.

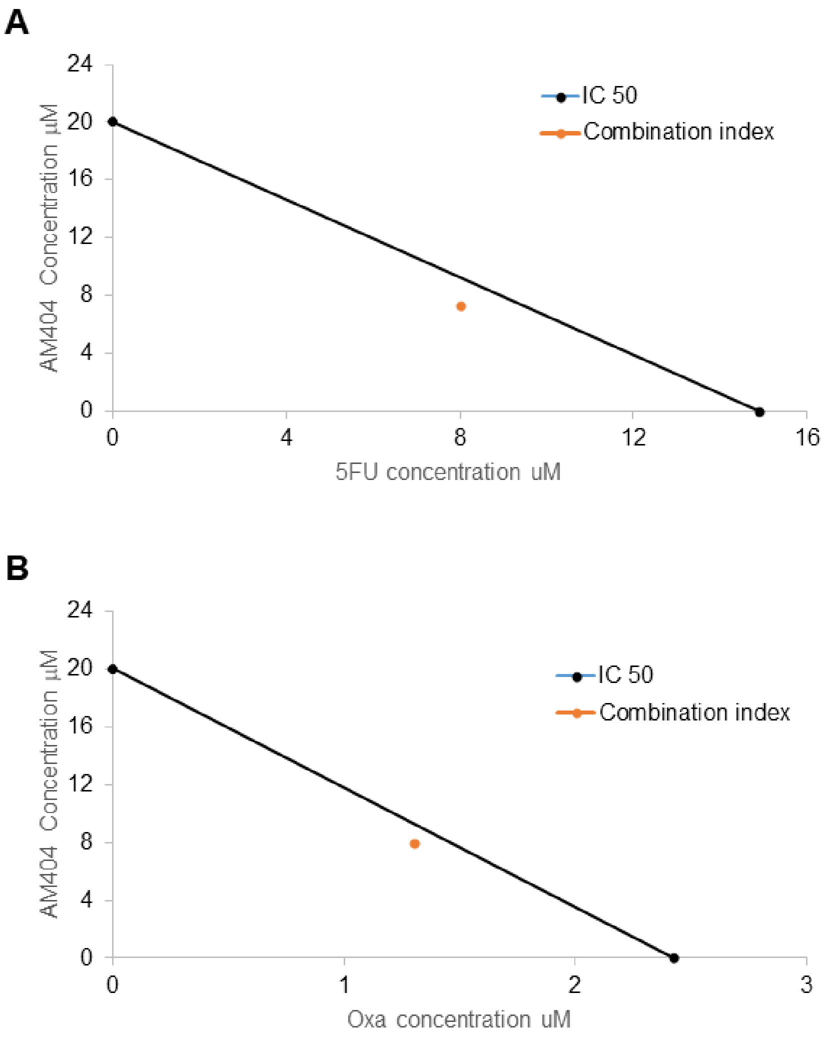


**Figure S2.** Isobologram analysis at the combination effect. **(A)** Combination therapy of AM404 and 5FU with combination index of 0.89. **(B)** Combination therapy of AM404 and Oxa with combination index of 0.94.

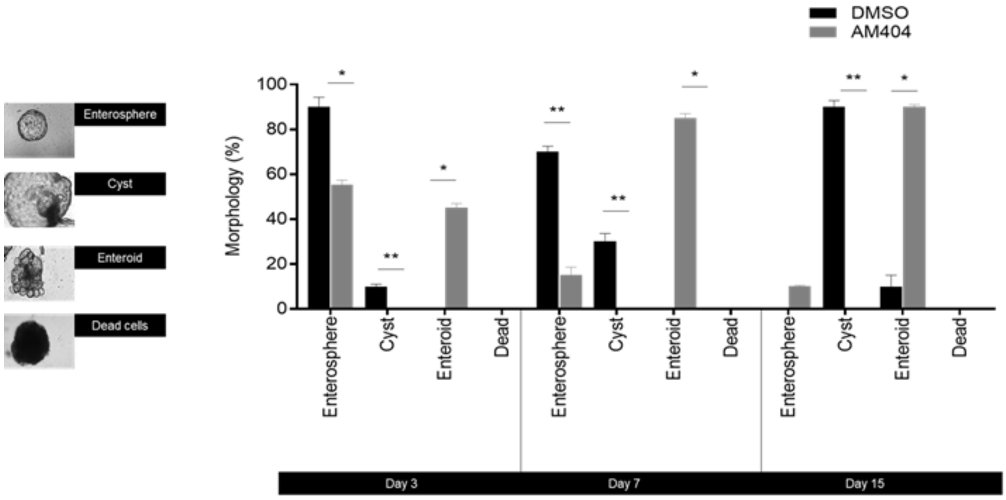


**Figure S3.** AM404 treatment presents enteroid-like structures and induces differentiation in organoids. Percentage of enterosphere, cyst, enteroids and dead cells at day 3, 7 and 15 days of treatment. Bars represent the mean of three different experiments ± SEM; * $p < 0.05$, ** $p < 0.01$, *** $p < 0.001$, using Student's t test.

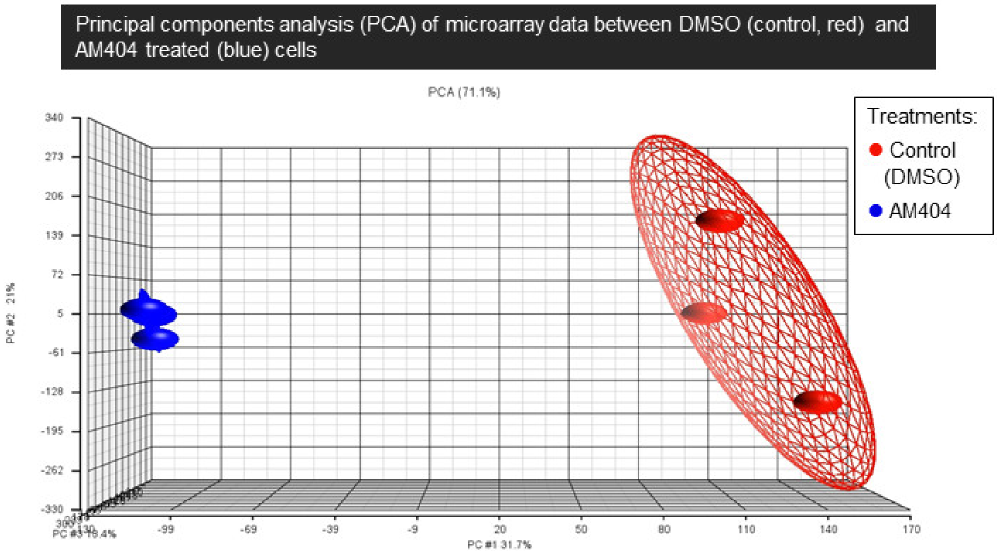


**Figure S4.** Principal components analysis (PCA) of microarray data between DMSO (control, red) and AM404 treated (blue) cells transcriptomes.

**A**

| Gene ontology (GO) biological pathways |
|---|
| Negative regulation of transcription from RNA polymerase II promoter (GO: 0000122) |
| DNA damage induced protein phosphorylation (GO: 0006975) |
| DNA repair (GO: 0006281) |
| Protein ubiquitination involved in ubiquitin-dependent protein catabolic process (GO: 0042787) |
| Reduction of transcription involved in G2./M transition of mitotic cell cycle (GO: 0000117) |
| Negative regulation of G2/S transition of mitotic cell cycle by negative regulation of transcription from RNA polymerase II |
| Ciliary basal body plasma membrane docking (GO: 0097711) |
| Histone H4 acetylation involved in response to DNA damage stimulus (GO: 20000776) |
| Regulation of transcription involved in G1/S transition of mitotic cell cycle (GO: 0000083) |
| Telomere maintenance in response to DNA damage (GO: 0043247) |

**B**

Genetic and physical interaction network of differentially expressed genes by AM404

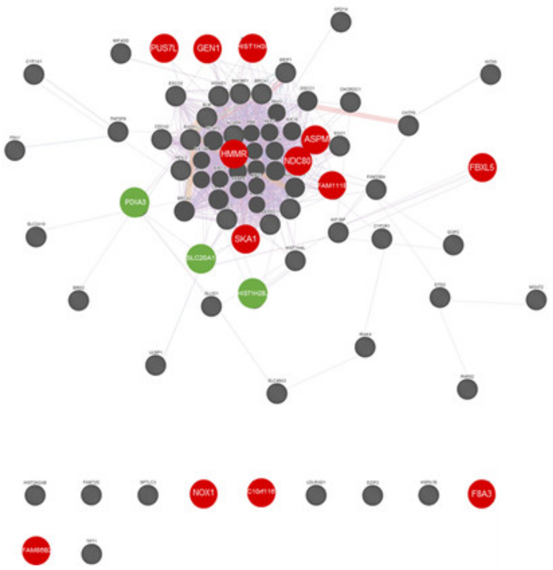


**Figure S5.** (**A**) Gene Ontology (GO) biological pathways for the 323 genes (mean ≥ 2 fold change) that had significantly altered their expression by AM404 treatment. Results were obtained using DAVID Bioinformatics Resources 6.8. (**B**) Interaction network of 75 differentially expressed genes (mean ≥2.5 fold change) by AM404 treatment. The GeneMANIA plug-in for Cytoscape was used to generate an interaction network based on previously documented interactions. Red and green nodes mark down- and upregulated 16 genes amongst other genes. .

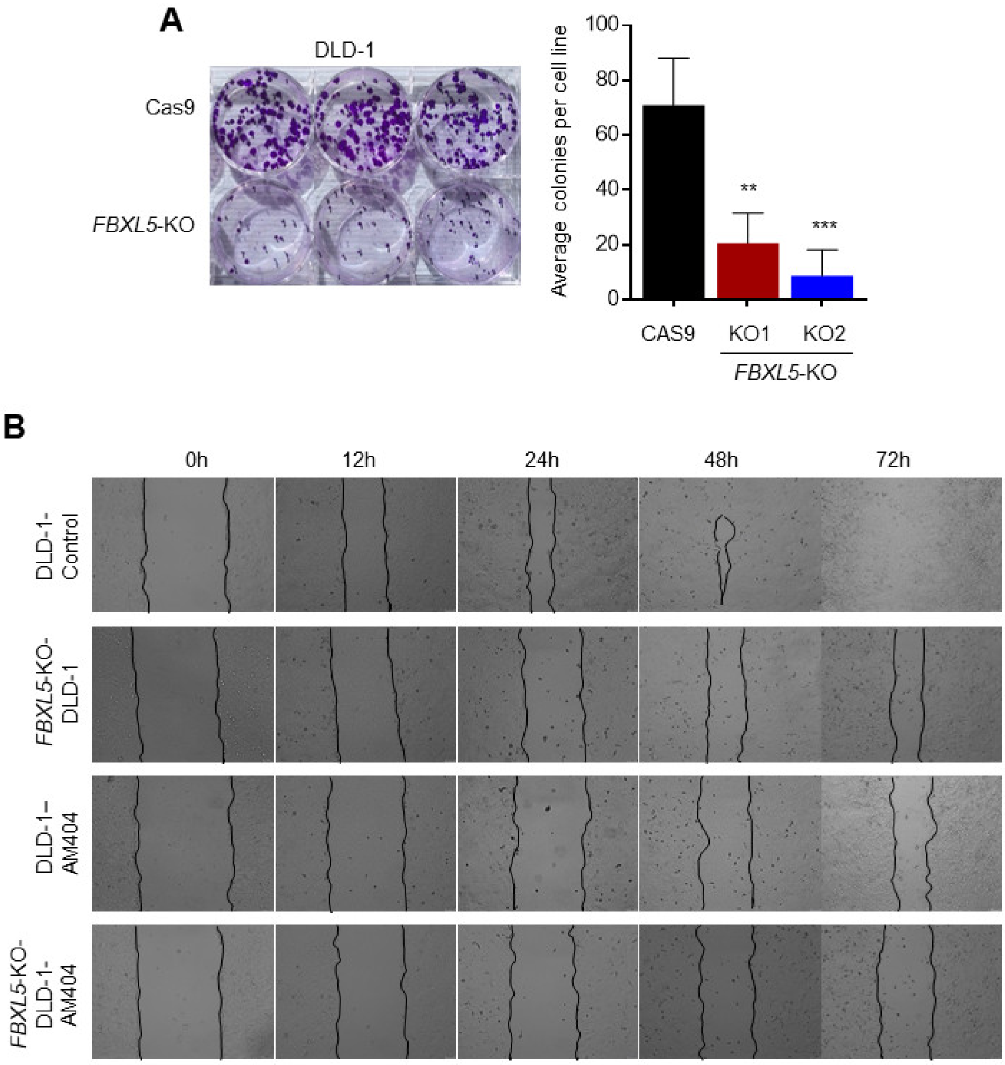


**Figure S6.** (**A**) Colony forming efficiency assays of DLD-Cas9 (control) and DLD-Cas9:FBXL5-gRNAs (CRISPR-knockout) cell lines (KO1 and KO2). Bars are expressed as mean ± S.E.M. ($n = 3$). **, $p < 0.01$; ***, $p < 0.001$. (**B**) Representative images of wound healing assay of DLD-Cas9 (control) and DLD-Cas9:FBXL5-gRNAs (CRISPR-knockout) cell lines treated with DMSO (control) and AM404 were taken at 0, 12, 24, 48 and 72 hours with phase-contrast microscope for quantification in triplicate samples as shown in Figure. 6E.

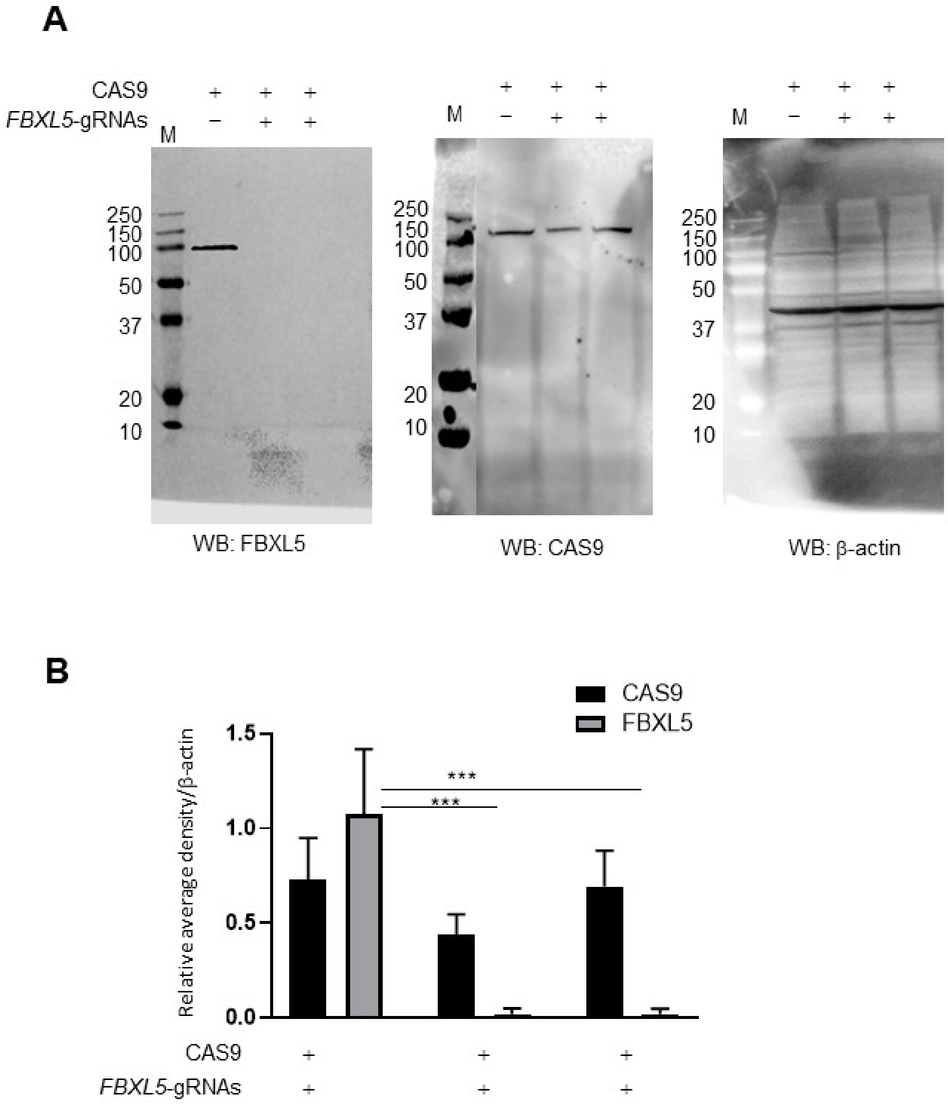


**Figure S7. (A)** Whole western blot images of Figure 6B. **(B)** FBXL5 and Cas9 intensity ratio versus -actin of each band showed in Figure 6B. Bars represent the mean of three different experiments ± SEM; ** $p < 0.01$, *** $p < 0.001$, using Student's $t$ test.

**Table S1.** List of primers and their sequences used for qRT-PCR assay.

| | | |
|---|---|---|
| CD44 | Forward | AGAAGGTGTGGGCAGAAGAA |
| | Reverse | AAATGCACCATTTCCTGAGA |
| NANOG | Forward | GCAGGCAACTCACTTTATCCCA |
| | Reverse | GGCAGCTTTAAGACTTTTCTGG |
| LGR5 | Forward | GACAACAGCAGTATGGACG |
| | Reverse | GCATTACAAGTAAGTGCCAG |
| OCT4 | Forward | AAGCTGCGGCCCTTGCTGCAG |
| | Reverse | AGCCCAAGCTGCTGGGCGATG |
| Krt20 | Forward | TGAAGAGCTGCGAAGTCAGAT |
| | Reverse | TCCTCTCTCAGTCTCATACTTCAGTC |
| CDX2 | Forward | AGCCAAGTGAAAACCAGGAC |
| | Reverse | CCAGATTTTAACCTGCCTCTCA |
| C-Jun | Forward | ACCCCAAGATCCTGAAACAG |
| | Reverse | ATCAGGCGCTCCAGCTCG |
| C-Myc | Forward | GGAGACACCGCCCACCA |
| | Reverse | GCGCTGCGTAGTTGTGCTG |

| | | |
|---|---|---|
| CCND1 | Forward | GCTGGAGGTCTGCGAGGA |
| | Reverse | CATCTTAGAGGCCACGAACA |
| CTNNB1 | Forward | TATTACGACAGACTGCC |
| | Reverse | TGAAGTCCTAAAGCTTG |
| CXCR4 | Forward | TTCTACCCCAATGACTTGTG |
| | Reverse | ATGTAGTAAGGCAGCCAACA |
| BMI-1 | Forward | CCAGGGCTTTTCAAAAATGA |
| | Reverse | CCGATCCAATCTGTTCTGGT |
| N-Cadherin | Forward | GACAATGCCCCTCAAGTGTT |
| | Reverse | CCATTAAGCCGAGTGATGGT |
| Vimentin | Forward | GAGAACTTTGCCGTTGAAGC |
| | Reverse | TCCAGCAGCTTCCTGTAGGT |
| FBXW7 | Forward | GAGACTTCATCTCCTTGCTTCCTAAA |
| | Reverse | CGCTTGCAGCAGGTCTTTG |
| B-actin | Forward | GCGCGGCTACAGCTTCA |
| | Reverse | CTTAATGTCACGCACGATTTCC |

**Table S2.** List of drugs selected during different stages of the screening.

| Primary Screening (50 drugs) | Re-screening | | | Common Compounds (11 Drugs) |
|---|---|---|---|---|
| | HCT116 (16 Drugs) | DLD-1 (11 Drugs) | SW480 (11 Drugs) | |
| 3,5,3′-Triiodothyronine<br>5-Nonyloxytryptamine HCl<br>Acitretin<br>AM 404<br>Benidipine HCl<br>Bifemelane HCl<br>Calcitriol<br>CGS 15943<br>Chloroxine<br>(+)-cis-Diltiazem HCl<br>Clofazimine<br>Dactinomycin<br>Daunorubicin HCl<br>Doxorubicin HCl<br>Ebselen<br>Efavirenz<br>Epigallocatechin gallate<br>Epirubicin HCl<br>Fenoldopam mesylate<br>Galanthamine HBr<br>Hexachlorophene<br>Honokiol<br>Icariin<br>Idarubicin HCl<br>Idebenone<br>Lacidipine<br>Methyldopa<br>MK 886<br>Mitoxantrone HCl<br>Nicotinamide<br>Nisoldipine<br>Oligomycin A<br>Oxytetracycline HCl<br>PD 81723<br>Pefloxacin mesylate | 5-Nonyloxytryptamine HCl<br>AM 404<br>Epirubicin HCl<br>Dactinomycin<br>Daunorubicin HCl<br>Doxorubicin HCl<br>Hexachlorophene<br>Honokiol<br>Idarubicin HCl<br>MK 886<br>Mitoxantrone HCl<br>Oligomycin A<br>Rifabutin<br>Rifampicin<br>Rifapentine<br>Zafirlukast | AM 404<br>Daunorubicin HCl<br>Doxorubicin HCl<br>Epirubicin HCl<br>Hexachlorophe ne<br>Honokiol<br>Idarubicin HCl<br>Itraconazole<br>MK 886<br>Rifabutin<br>Rifapentine | AM 404<br>Daunorubicin HCl<br>Doxorubicin HCl<br>Epirubicin HCl<br>Hexachlorophene<br>Honokiol<br>Idarubicin HCl<br>Itraconazole<br>MK 886<br>Rifabutin<br>Rifapentine | AM 404<br>Daunorubicin HCl<br>Epirubicin HCl<br>Hexachlorophene<br>Honokiol<br>Idarubicin HCl<br>Itraconazole<br>MK 886<br>Rifabutin<br>Rifapentine |

| |
|---|
| Phylloquinone |
| Pyrazinamide |
| Raloxifene HCl |
| Rifabutin |
| Rifampicin |
| Rifapentine |
| Rolitetracycline |
| Symmetrel |
| Taxifolin-(+/−) |
| Tolterodine tartrate |
| Trans-Retinoic acid |
| Tremulacin |
| Trimebutine maleate |
| Vinorelbine tartrate |
| Zafirlukast |

**Table S3.** List of differentially expressed genes (mean ≥2 fold change) in AM404 treated colonospheres.

| Gene Symbol | Fold-Change |
|---|---|
| SLC20A1 | 3.40922 |
| SNORA31 | 3.23582 |
| SNORA28 | 3.13565 |
| MIR3115 | 3.11244 |
| HIST1H2BJ | 3.09949 |
| MIR637 | 2.80158 |
| SPDYA | 2.77065 |
| TNFSF9 | 2.74608 |
| CHORDC1 | 2.71431 |
| SNORD59B | 2.68054 |
| TPT1 | 2.67106 |
| OTTHUMG00000165727 | 2.57599 |
| BRD2 | 2.5751 |
| GLUD1 | 2.54969 |
| MIR3662 | 2.54056 |
| HSPA1B | 2.53474 |
| HIST2H2BE | 2.40498 |
| BHLHE40 | 2.40393 |
| FDPS | 2.38535 |
| HSPA1B | 2.36471 |
| SNORA10 | 2.35812 |
| HSPA1B | 2.35085 |
| MPZL3 | 2.3379 |
| IL8 | 2.32806 |
| HSPA1A | 2.32556 |
| SNORD51 | 2.32159 |
| CTGF | 2.30568 |
| HSPA1B | 2.29133 |
| KLF6 | 2.27076 |
| HMGCS1 | 2.26364 |
| LOC100129785 | 2.26142 |
| TSC22D3 | 2.26104 |
| LOC100506766 | 2.25716 |
| OTTHUMG00000171211 | 2.24005 |
| PPP1R2 | 2.22361 |
| HSPA1A | 2.19472 |
| LOC554174 | 2.1828 |

| | |
|---|---|
| ZBTB40-IT1 | 2.16383 |
| TMEM55B | 2.14921 |
| MAFG | 2.14053 |
| PRPF39 | 2.12726 |
| PER1 | 2.11071 |
| INSIG1 | 2.09923 |
| ZBTB34 | 2.08724 |
| ECM1 | 2.067 |
| MIR3143 | 2.05838 |
| ADM | 2.05594 |
| GRHL1 | 2.05407 |
| JUN | 2.05354 |
| MVD | 2.05347 |
| CLK1 | 2.05199 |
| HIST1H4B | 2.04491 |
| SNORD58A | 2.03921 |
| RNF39 | 2.03291 |
| YOD1 | 2.02659 |
| ARL14 | 2.02412 |
| NR4A2 | 2.02206 |
| CDKN1C | 2.01733 |
| LRCH4 | 2.0168 |
| RPLP1 | 2.01204 |
| OTTHUMG00000154962 | 2.00453 |
| FANCD2 | −2.00047 |
| ACADM | −2.00231 |
| OMA1 | −2.00622 |
| IRAK1BP1 | −2.00897 |
| FASTKD3 | −2.01007 |
| CCP110 | −2.01069 |
| NCAPG | −2.01211 |
| LAMP3 | −2.01324 |
| SEPHS2 | −2.01373 |
| MLF1IP | −2.01385 |
| CCDC71 | −2.01528 |
| KIF20A | −2.01628 |
| HENMT1 | −2.01765 |
| SNORD72 | −2.01799 |
| H2AFY2 | −2.01904 |
| ADAT1 | −2.01967 |
| SULT1C2 | −2.02463 |
| TIRAP | −2.02682 |
| C12orf66 | −2.0271 |
| MINA | −2.02816 |
| UBLCP1 | −2.0351 |
| ZNF594 | −2.03544 |
| RFC5 | −2.03811 |
| DSCC1 | −2.03918 |
| UEVLD | −2.03929 |
| KLK10 | −2.03937 |
| CEP19 | −2.04253 |
| ID3 | −2.04304 |
| DHODH | −2.04324 |
| FECH | −2.04569 |
| SELRC1 | −2.0464 |
| YEATS4 | −2.04749 |

| | |
|---|---|
| TTC26 | −2.04789 |
| SFXN2 | −2.05314 |
| TRIM32 | −2.0533 |
| DLAT | −2.05431 |
| FAM114A2 | −2.05534 |
| DSTNP2 | −2.05573 |
| GPR1 | −2.0561 |
| FJX1 | −2.05917 |
| BUB1 | −2.06027 |
| LOC100129502 | −2.06418 |
| SKAP1 | −2.06454 |
| GSG2 | −2.06499 |
| KIAA0586 | −2.06713 |
| USP13 | −2.06889 |
| POLI | −2.06894 |
| KIAA1107 | −2.07024 |
| DCLRE1A | −2.07025 |
| HCG15 | −2.07153 |
| ERAL1 | −2.07185 |
| PLK4 | −2.07297 |
| CABYR | −2.07395 |
| AASDHPPT | −2.07559 |
| LOC100507449 | −2.07571 |
| KLHL4 | −2.07778 |
| CCDC113 | −2.07969 |
| RBL1 | −2.08169 |
| ASF1B | −2.0852 |
| SKA3 | −2.10319 |
| TICRR | −2.10426 |
| WDR92 | −2.1047 |
| FXYD3 | −2.10478 |
| SYK | −2.10558 |
| TBCK | −2.10995 |
| RPGRIP1L | −2.11011 |
| HAUS1 | −2.11344 |
| TRMT61B | −2.11432 |
| CENPK | −2.11466 |
| DNAJC11 | −2.12161 |
| TRMT1L | −2.1234 |
| CASP6 | −2.12733 |
| SNORD111 | −2.13249 |
| OSGEPL1 | −2.13254 |
| TMEM9B-AS1 | −2.13855 |
| SNORD100 | −2.1433 |
| CDK1 | −2.14947 |
| FAM173B | −2.15377 |
| THEM4 | −2.15445 |
| ASCL2 | −2.15581 |
| SIRT5 | −2.15946 |
| ZMYND11 | −2.16142 |
| MBLAC2 | −2.16328 |
| IGFL4 | −2.16683 |
| MRPL15 | −2.16767 |
| KLHDC1 | −2.16798 |
| FARSB | −2.16817 |
| NMRK1 | −2.17103 |

| | |
|---|---|
| DYNC2LI1 | −2.1717 |
| PIGV | −2.17174 |
| ZNF280B | −2.18593 |
| SLC25A10 | −2.19245 |
| C2orf43 | −2.19248 |
| PRSS35 | −2.20022 |
| ERCC4 | −2.20079 |
| MOCOS | −2.20219 |
| CPT2 | −2.204 |
| PNPO | −2.20535 |
| SLC16A13 | −2.21061 |
| ANKEF1 | −2.21115 |
| GTF2H3 | −2.21185 |
| TRIP11 | −2.21495 |
| KIF23 | −2.2182 |
| TST | −2.22501 |
| ZFP69 | −2.22759 |
| COX10-AS1 | −2.22822 |
| PLK1 | −2.22996 |
| C6orf70 | −2.23309 |
| C5orf63 | −2.23424 |
| ZNF266 | −2.23441 |
| RRS1 | −2.2374 |
| C4orf32 | −2.2399 |
| KIAA1524 | −2.24724 |
| DET1 | −2.24786 |
| RMI1 | −2.25043 |
| CDX2 | −2.25241 |
| SCRN3 | −2.25434 |
| PLA2G7 | −2.25701 |
| PRMT3 | −2.25733 |
| LOC100506963 | −2.25794 |
| SBSPON | −2.26796 |
| SNORD9 | −2.26939 |
| C6orf203 | −2.26952 |
| POLQ | −2.27153 |
| ZNF554 | −2.27756 |
| PPIL1 | −2.28812 |
| KRCC1 | −2.28819 |
| MRM1 | −2.28824 |
| NARG2 | −2.29049 |
| LCN2 | −2.29312 |
| THAP10 | −2.29872 |
| CETN3 | −2.29988 |
| MCM10 | −2.30303 |
| HJURP | −2.30314 |
| OBSCN | −2.30829 |
| HSD17B1 | −2.30857 |
| LOC202181 | −2.30882 |
| PMS2 | −2.31583 |
| SRBD1 | −2.31753 |
| KIF11 | −2.31855 |
| XRCC2 | −2.31985 |
| RBM45 | −2.32138 |
| ZBED3 | −2.32373 |
| CCDC125 | −2.32511 |

| | |
|---|---|
| SLC9A8 | −2.32792 |
| TMEM52B | −2.3406 |
| CKAP2L | −2.34853 |
| GTF2IRD2 | −2.35277 |
| LOC100507516 | −2.35412 |
| KIAA1377 | −2.35468 |
| FAM83D | −2.35958 |
| MARS2 | −2.36325 |
| NCOA5 | −2.36498 |
| TSEN2 | −2.36718 |
| KIF20B | −2.37419 |
| FKTN | −2.3758 |
| SDAD1 | −2.3768 |
| SNORD126 | −2.37717 |
| STRADA | −2.38501 |
| IFT80 | −2.38685 |
| FAM86DP | −2.38812 |
| LYRM7 | −2.38851 |
| CHAF1B | −2.39505 |
| OTTHUMG00000059903 | −2.39824 |
| PRIM1 | −2.39825 |
| PSMD5-AS1 | −2.40089 |
| SNORD69 | −2.40203 |
| CSRP2BP | −2.40465 |
| BRCA2 | −2.40876 |
| CFTR | −2.41427 |
| GEMIN4 | −2.41471 |
| CENPF | −2.42261 |
| ZFP90 | −2.42381 |
| RWDD2B | −2.4249 |
| MGME1 | −2.42706 |
| SYCE2 | −2.44092 |
| C12orf4 | −2.44353 |
| OTTHUMG00000037424 | −2.44592 |
| FLJ14186 | −2.44863 |
| ZNF252P | −2.4538 |
| ACER2 | −2.45416 |
| CDC25C | −2.45885 |
| PTCD2 | −2.46617 |
| ABO | −2.46676 |
| GINS1 | −2.46788 |
| ZNF23 | −2.47319 |
| CES2 | −2.48362 |
| CLUAP1 | −2.48464 |
| USP40 | −2.49191 |
| RNF170 | −2.49736 |
| WDR76 | −2.49784 |
| CYP2R1 | −2.5001 |
| LDLRAD1 | −2.51564 |
| SLC2A10 | −2.52026 |
| SHCBP1 | −2.5219 |
| MGAT2 | −2.53704 |
| CDC7 | −2.57597 |
| NEIL3 | −2.57773 |
| BRCA1 | −2.58799 |
| KIAA1279 | −2.58836 |

| | |
|---|---|
| FAM72D | −2.60089 |
| FAM86HP | −2.60142 |
| NCAPH | −2.61352 |
| MIF4GD | −2.61989 |
| WDHD1 | −2.6237 |
| SGOL1 | −2.63519 |
| GOPC | −2.65037 |
| PARS2 | −2.65689 |
| BLM | −2.67003 |
| FAM208A | −2.67272 |
| F8A1 | −2.68143 |
| SNORD121A | −2.69081 |
| CYP1A1 | −2.69622 |
| RAD51 | −2.73685 |
| SLC46A3 | −2.7375 |
| CHTF8 | −2.74581 |
| ESCO2 | −2.74641 |
| DZIP3 | −2.74818 |
| SPTLC3 | −2.76889 |
| IRAK4 | −2.77648 |
| DTD2 | −2.78523 |
| SNORD90 | −2.79634 |
| OTTHUMG00000163993 | −2.80351 |
| SNORD63 | −2.81223 |
| DTL | −2.81506 |
| CDCA2 | −2.85031 |
| HIST1H4L | −2.85568 |
| LINC00858 | −2.87467 |
| HIST2H2AB | −2.88008 |
| DEPDC1 | −2.94581 |
| INTS5 | −2.94694 |
| ULBP1 | −2.99393 |
| F8A3 | −2.99583 |
| BRIP1 | −2.99929 |
| FBXL5 | −3.0001 |
| FAM111B | −3.06187 |
| HMMR | −3.06684 |
| NOX1 | −3.09866 |
| PDIA3 | −3.20377 |
| FAM86B2 | −3.22522 |
| F8A3 | −3.26485 |
| MIR1184-1 | −3.26485 |
| C1orf116 | −3.41795 |
| ASPM | −3.44786 |
| SKA1 | −3.45407 |
| NDC80 | −3.46322 |
| PUS7L | −3.50814 |
| SNORA2A | −3.6422 |
| GEN1 | −3.64797 |
| HIST1H3I | −3.89911 |

© 2020 by the authors. Licensee MDPI, Basel, Switzerland. This article is an open access article distributed under the terms and conditions of the Creative Commons Attribution (CC BY) license (http://creativecommons.org/licenses/by/4.0/).
